# Supplementary material for: Admission photoplethysmography-based mortality prediction in hospitalized Ugandan children with suspected or confirmed infection: A feasibility study
Source: PLOS Digit Health. 2026 Aug 6;5(8):e0001056. doi: 10.1371/journal.pdig.0001056 (PMC13446674; doi:10.1371/journal.pdig.0001056)
Supplement: Supplementary file 2 [file pdig.0001056.s002.docx]

**S2 Appendix. PPG-derived features and definitions.**

**Contents**

[Table 1. Examples of PPG-derived features grouped by extraction source.	2](#_Toc209949438)

[Fig 1. Examples of features extracted from PPG and its second-order derivative.	4](#_Toc209949439)

**Table 1. Examples of PPG-derived features grouped by extraction source.**

| **Feature, *unit*** | **Feature description, *formula*** |
| --- | --- |
| **Source: PPG** | |
| pulseTime, *ms* | Pulse interval, time from onset to end, $t_{end}-t_{onset}$ |
| crestTime, *ms* | Crest time (or risetime), time from onset to systolic peak, $t_{sys}-t_{onset}$ |
| cataTime, *ms* | Time from systolic peak to end, $t_{end}-t_{sys}$ |
| sysTime, *ms* | Time in systolic phase from onset to dicrotic notch, $t_{dic}-t_{onset}$ |
| diasTime, *ms* | Time in diastolic phase from dicrotic notch to end, $t_{end}-t_{dic}$ |
| deltaTime, *ms* | Time delay between systolic and diastolic peaks, $t_{dia}-t_{sys}$ |
| SysDiasTimeRatio | Ratio of sysTime to diasTime, ${{(t}_{dic}-t_{onset})}/{{(t}_{end}-t_{dic})}$ |
| deltaTimeRel | Ratio of deltaTime to pulseTime, ${(t_{dia}-t_{sys})}/{(t_{end}-t_{onset})}$ |
| crestTimeRel | Ratio of crestTime to sysTime, ${(t_{sys}-t_{onset})}/{(t_{dic}-t_{onset})}$ |
| W25 (W50), *ms* | Pulse width at 25% (50%) of systolic peak amplitude |
| sysAmp, *a.u.* | Amplitude of systolic peak from onset, $x_{sys}-x_{onset}$ |
| dicAmp, *a.u.* | Amplitude of dicrotic notch from onset, $x_{dic}-x_{onset}$ |
| diasAmp, *a.u.* | Amplitude of diastolic peak from onset, $x_{dia}-x_{onset}$ |
| SysDiasAmpDiff, *a.u.* | Amplitude difference between systolic and diastolic peaks, $x_{sys}-x_{dia}$ |
| RI | Reflection index, ${(x_{dia}-x_{onset})}/{(x_{sys}-x_{onset})}$ |
| AI | Augmentation index, ${(x_{sys}-x_{dia})}/{(x_{sys}-x_{onset})}$ |
| pulseHeight, *a.u.* | Amplitude difference between max and min of pulse, $x_{max}-x_{min}$ |
| pulseArea, *ms·a.u.* | Area bounded by times of onset ($t_{onset}$) and end ($t_{end}$) |
| area1, *ms·a.u.* | Area bounded by times of onset ($t_{onset}$) and dicrotic notch ($t_{dic}$) |
| area2, *ms·a.u.* | Area bounded by times of dicrotic notch ($t_{dic}$) and end ($t_{end}$) |
| IPA | Inflection point area ratio, ${{area}_{2}}/{{area}_{1}}$ |
| STT, *a.u./ms* | Slope transit time, slope of straight line between onset and systolic peak |
| skewPulse | Pulse skewness, asymmetry of pulse waveform around the mean |
| kurtPulse | Pulse kurtosis, peakedness or flatness of pulse waveform, indicating sharpness |
| **Source: 2^nd^ derivative of PPG** | |
| abTime, *ms* | Time delay between a-peak and b-peak, $t_{b}-t_{a}$ |
| a, *a.u.* | Amplitude of a-peak, $x_{a}$ |
| b, *a.u.* | Amplitude of b-peak, $x_{b}$ |
| e, *a.u.* | Amplitude of e-peak, $x_{e}$ |
| b-a, *a.u.* | Amplitude difference between a-peak and b-peak, $x_{b}-x_{a}$ |
| b/a | Ratio of b-peak amplitude to a-peak amplitude, ${x_{b}}/{x_{a}}$ |
| e/a | Ratio of e-peak amplitude to a-peak amplitude, ${x_{e}}/{x_{a}}$ |
| (b-e)/a | Vascular aging index when c- and d-peaks are missing, ${(x_{b}-x_{e})}/{x_{a}}$ |
| abSlope, *a.u./ms* | Slope of straight line between a-peak and b-peak, ${(x_{b}-x_{a})}/{abTime}$ |
| **Source: PPI series** | |
| Pulse rate, *bpm* | Number of pulses per minute, ${60}/{PPI}$ |
| SDPP, *ms* | Standard deviation of PPIs |
| medPP, *ms* | Median of PPIs |
| madPP, *ms* | Median absolute deviation of PPIs, $median(\left\vert{PPI}_{i}-median\left( PPI \right) \right\vert)$ |
| tprPP | Turning point ratio of PPIs, $\frac{\#Points where PPI > 0}{\#Points in PPI}$ |
| skewPP | Skewness of PPIs distribution |
| kurtPP | Kurtosis of PPIs distribution |
| SDSD, *ms* | Standard deviation of consecutive PPI differences |
| RMSSD, *ms* | Root mean square of consecutive PPI differences, $\sqrt{\frac{1}{N-1}\sum_{i=1}^{N-1} \left\vert{PPI}_{i+1}-{PPI}_{i} \right\vert^{2}}$ |
| tprPPdiff | Turning point ratio of consecutive PPI differences |
| pNN20 (pNN50), *%* | Percentage of consecutive PPIs that differ by more than 20 ms (50 ms) |
| SD1, *ms* | Standard deviation perpendicular to the line of identity (or along the minor axis) in Poincaré plot |
| SD2, *ms* | Standard deviation along the line of identity (or along the major axis) in Poincaré plot |
| SD1/SD2 | Ratio of SD1 to SD2 |
| VLF, *ms^2^* | Absolute power of very-low-frequency band (0.0033–0.04 Hz) |
| LF, *ms^2^* | Absolute power of low-frequency band (0.04–0.2 Hz for newborns, 0.04–0.15 Hz for infants) |
| HF, *ms^2^* | Absolute power of high-frequency band (0.20–2.00 Hz for newborns, 0.20–1.40 Hz for infants) |
| LF/HF | Ratio of LF to HF |
| LFn | Normalized power in low-frequency band (0.04–0.2 Hz for newborns, 0.04–0.15 Hz for infants) |
| HFn | Normalized power in high-frequency band (0.20–2.00 Hz for newborns, 0.20–1.40 Hz for infants) |
| entropyPP | Shannon entropy, unpredictability of PPIs |
| **Source: Pulse oximeter** | |
| SpO_2_, *%* | Oxygen saturation |
| meanPERF | Mean of perfusion index |
| medPERF | Median of perfusion index |
| stdPERF | Standard deviation of perfusion index |

**Fig 1. Examples of features extracted from PPG and its second-order derivative.**
