# Supplementary material for: Admission photoplethysmography-based mortality prediction in hospitalized Ugandan children with suspected or confirmed infection: A feasibility study
Source: PLOS Digit Health. 2026 Aug 6;5(8):e0001056. doi: 10.1371/journal.pdig.0001056 (PMC13446674; doi:10.1371/journal.pdig.0001056)
Supplement: Supplementary file 3 [file pdig.0001056.s003.docx]

**S3 Appendix. Hyperparameter search spaces for each model.**

**Table 1. Model-specific hyperparameter search spaces.**

| **Model** | **Hyperparameter** | **Search space** |
| --- | --- | --- |
| LR | *penalty* | ['elasticnet'] |
|  | *solver:* algorithm of optimization problem | ['saga'] |
|  | *C:* inverse of regularization strength | numpy.logspace(-4,4,num=50) |
|  | *l1_ratio:* Elastic-Net mixing parameter | numpy.arange(0,0.91,0.01) |
| RF | *n_estimators:* # of trees in forest | [10,100,1000] |
|  | *max_features:* # of features when looking for best split | ['sqrt','log2'] |
|  | *max_depth:* maximum depth of tree | [2,3] |
|  | *min_samples_leaf:* minimum # of samples required at leaf node | [12,14,16] |
| XGB | *n_estimators:* # of boosting rounds | [100,200,300] |
|  | *max_depth:* maximum tree depth for base learners | [3,4,5] |
|  | *learning_rate:* boosting learning rate | [0.01,0.1,0.2] |
|  | *subsample:* subsample ratio of training instance | [0.7,0.8,0.9] |
|  | *colsample_bytree:* subsample ratio of columns when building a tree | [0.7,0.8,0.9] |
|  | *gamma:* minimum loss reduction for making partition on leaf node | [0,0.1,0.2,0.3] |
|  | *reg_alpha:* L1 regularization term on weights | [0,0.01,0.1,1] |
|  | *reg_lambda:* L2 regularization term on weights | [1,2,3,4] |
| MLP | *hidden_layer_sizes:* # of neurons in i^th^ hidden layer | [(50,),(100,),(150,)] |
|  | *learning_rate_init:* initial learning rate | numpy.linspace(0.001,0.01,5) |
|  | *learning_rate:* learning rate schedule for weight updates | ['constant','adaptive'] |
|  | *activation:* activation function for hidden layer | ['relu','tanh'] |
|  | *alpha:* strength of L2 regularization term | numpy.linspace(0.0001,0.001,5) |
|  | *solver:* solver for weight optimization | ['adam','sgd'] |

Detailed hyperparameter definitions are available in the scikit-learn documentation: <https://scikit-learn.org/stable/>.
