# Supplementary material for: Admission photoplethysmography-based mortality prediction in hospitalized Ugandan children with suspected or confirmed infection: A feasibility study
Source: PLOS Digit Health. 2026 Aug 6;5(8):e0001056. doi: 10.1371/journal.pdig.0001056 (PMC13446674; doi:10.1371/journal.pdig.0001056)
Supplement: Supplementary file 4 [file pdig.0001056.s004.docx]

**S4 Appendix. Excluded population characteristics.**

**Table 1. Baseline characteristics of the excluded population, stratified by age cohort.**

|  | **0–6-month cohort** | | **6–60-month cohort** | |
| --- | --- | --- | --- | --- |
|  | **(n = 274)** | | **(n = 390)** | |
| **Variable** | **n (%) or Median [Q1,Q3]** | **n Missing (%)** | **n (%) or Median [Q1,Q3]** | **n Missing (%)** |
| **A) Discharge characteristics** | | | | |
| Outcome |  | 0 (0%) |  | 0 (0%) |
| Survivors | 230 (83.9%) |  | 353 (90.5%) |  |
| Non-survivors | 44 (16.1%) |  | 37 (9.5%) |  |
| *Died in hospital* | 42 (95.4%) |  | 36 (97.3%) |  |
| *Died on discharge day* | 2 (4.6%) |  | 0 (0%) |  |
| *Died within 48hrs post-discharge* | 0 (0%) |  | 1 (2.7%) |  |
| Length of stay, days |  | 0 (0%) |  | 0 (0%) |
| Survivors | 5 [3,6] |  | 4 [2,6] |  |
| Non-survivors | 1 [1,2] |  | 1 [0,3] |  |
| **B) Demographics** | | | | |
| Sex, male | 150 (54.7%) | 0 (0%) | 205 (52.6%) | 0 (0%) |
| Age, months | 1.9 [0.5,3.6] | 0 (0%) | 17.7 [11,27] | 0 (0%) |
| **C) Admission anthropometry** | | | | |
| Weight-for-age z-score | -1.2 [-2.5,-0.2] | 0 (0%) | -1 [-2.2,-0.1] | 2 (0.5%) |
| < -3 | 53 (19.3%) |  | 60 (15.4%) |  |
| -3 to -2 | 33 (12%) |  | 60 (15.4%) |  |
| > -2 | 188 (68.6%) |  | 268 (68.7%) |  |
