# Supplementary material for: Admission photoplethysmography-based mortality prediction in hospitalized Ugandan children with suspected or confirmed infection: A feasibility study
Source: PLOS Digit Health. 2026 Aug 6;5(8):e0001056. doi: 10.1371/journal.pdig.0001056 (PMC13446674; doi:10.1371/journal.pdig.0001056)
Supplement: Supplementary file 5 [file pdig.0001056.s005.docx]

**S5 Appendix. Sensitivity analysis of resampling techniques.**

**Table 1. Impact of resampling techniques on model performance metrics, stratified by age cohort.**

|  |  | **0–6-month cohort** | | | | **6–60-month cohort** | | | |
| --- | --- | --- | --- | --- | --- | --- | --- | --- | --- |
|  |  | **LR*_10_*** | **RF*_10_*** | **XGB*_10_*** | **MLP*_10_*** | **LR*_10_*** | **RF*_10_*** | **XGB*_10_*** | **MLP*_10_*** |
| AUROC | Baseline* | **0.67** (0.58-0.74) | **0.70** (0.62-0.77) | **0.70** (0.62-0.76) | **0.67** (0.58-0.75) | **0.65** (0.56-0.73) | **0.68** (0.60-0.75) | **0.67** (0.56-0.73) | **0.65** (0.57-0.73) |
|  | ROS | **0.67** (0.59-0.74) | **0.70** (0.62-0.75) | **0.68** (0.60-0.74) | **0.66** (0.58-0.74) | **0.65** (0.57-0.75) | **0.66** (0.57-0.74) | **0.65** (0.57-0.74) | **0.66** (0.59-0.71) |
|  | RUS | **0.66** (0.59-0.74) | **0.68** (0.62-0.73) | **0.66** (0.60-0.71) | **0.66** (0.59-0.74) | **0.65** (0.55-0.71) | **0.66** (0.60-0.75) | **0.66** (0.58-0.75) | **0.65** (0.57-0.71) |
|  | SMOTEENN | **0.66** (0.59-0.74) | **0.67** (0.60-0.74) | **0.65** (0.57-0.70) | **0.66** (0.58-0.74) | **0.65** (0.55-0.73) | **0.62** (0.54-0.68) | **0.63** (0.52-0.70) | **0.65** (0.55-0.72) |
| AUPRC | Baseline | **0.20** (0.12-0.28) | **0.19** (0.13-0.25) | **0.18** (0.12-0.27) | **0.20** (0.12-0.29) | **0.12** (0.06-0.19) | **0.13** (0.07-0.22) | **0.14** (0.06-0.22) | **0.12** (0.07-0.18) |
|  | ROS | **0.20** (0.12-0.28) | **0.18** (0.12-0.26) | **0.17** (0.11-0.25) | **0.19** (0.12-0.29) | **0.12** (0.05-0.17) | **0.12** (0.06-0.21) | **0.10** (0.06-0.15) | **0.11** (0.07-0.16) |
|  | RUS | **0.19** (0.11-0.27) | **0.18** (0.12-0.25) | **0.15** (0.12-0.21) | **0.19** (0.12-0.28) | **0.11** (0.07-0.18) | **0.12** (0.07-0.22) | **0.11** (0.06-0.16) | **0.12** (0.07-0.20) |
|  | SMOTEENN | **0.19** (0.12-0.27) | **0.17** (0.11-0.26) | **0.15** (0.10-0.22) | **0.19** (0.11-0.29) | **0.11** (0.06-0.16) | **0.09** (0.06-0.17) | **0.10** (0.05-0.16) | **0.11** (0.05-0.15) |
| Sensitivity | Baseline | **0.84** (0.82-0.88) | **0.83** (0.82-0.88) | **0.83** (0.82-0.85) | **0.83** (0.82-0.89) | **0.84** (0.82-0.89) | **0.83** (0.82-0.86) | **0.83** (0.82-0.84) | **0.83** (0.82-0.89) |
|  | ROS | **0.83** (0.82-0.89) | **0.83** (0.82-0.88) | **0.83** (0.82-0.85) | **0.84** (0.82-0.90) | **0.83** (0.82-0.86) | **0.83** (0.82-0.86) | **0.83** (0.82-0.84) | **0.83** (0.82-0.87) |
|  | RUS | **0.83** (0.82-0.85) | **0.83** (0.82-0.86) | **0.83** (0.82-0.85) | **0.83** (0.82-0.87) | **0.83** (0.82-0.90) | **0.83** (0.82-0.85) | **0.83** (0.82-0.87) | **0.83** (0.82-0.86) |
|  | SMOTEENN | **0.84** (0.82-0.92) | **0.83** (0.82-0.86) | **0.83** (0.82-0.84) | **0.83** (0.82-0.88) | **0.84** (0.82-0.94) | **0.83** (0.82-0.86) | **0.83** (0.82-0.86) | **0.83** (0.82-0.87) |
| Specificity | Baseline | **0.35** (0.16-0.55) | **0.44** (0.29-0.61) | **0.42** (0.26-0.55) | **0.35** (0.15-0.60) | **0.34** (0.17-0.49) | **0.39** (0.20-0.56) | **0.38** (0.21-0.51) | **0.35** (0.17-0.54) |
|  | ROS | **0.36** (0.15-0.50) | **0.43** (0.24-0.55) | **0.41** (0.29-0.51) | **0.34** (0.09-0.53) | **0.35** (0.16-0.53) | **0.37** (0.22-0.56) | **0.35** (0.21-0.54) | **0.35** (0.19-0.54) |
|  | RUS | **0.36** (0.18-0.55) | **0.40** (0.26-0.50) | **0.38** (0.23-0.51) | **0.35** (0.17-0.54) | **0.34** (0.19-0.50) | **0.38** (0.22-0.55) | **0.34** (0.17-0.54) | **0.34** (0.19-0.46) |
|  | SMOTEENN | **0.35** (0.16-0.54) | **0.41** (0.25-0.56) | **0.39** (0.25-0.50) | **0.36** (0.12-0.60) | **0.35** (0.21-0.52) | **0.34** (0.22-0.44) | **0.33** (0.19-0.48) | **0.34** (0.19-0.50) |
| PPV | Baseline | **0.09** (0.07-0.12) | **0.10** (0.08-0.14) | **0.10** (0.08-0.12) | **0.09** (0.07-0.13) | **0.05** (0.04-0.07) | **0.06** (0.04-0.07) | **0.05** (0.04-0.07) | **0.05** (0.04-0.07) |
|  | ROS | **0.09** (0.07-0.11) | **0.10** (0.08-0.12) | **0.10** (0.08-0.11) | **0.09** (0.07-0.12) | **0.05** (0.04-0.07) | **0.05** (0.04-0.07) | **0.05** (0.04-0.07) | **0.05** (0.04-0.07) |
|  | RUS | **0.09** (0.07-0.12) | **0.09** (0.08-0.11) | **0.09** (0.07-0.11) | **0.09** (0.07-0.12) | **0.05** (0.04-0.07) | **0.06** (0.04-0.07) | **0.05** (0.04-0.07) | **0.05** (0.04-0.06) |
|  | SMOTEENN | **0.09** (0.07-0.12) | **0.10** (0.08-0.12) | **0.09** (0.08-0.11) | **0.09** (0.07-0.13) | **0.05** (0.04-0.07) | **0.05** (0.04-0.06) | **0.05** (0.04-0.06) | **0.05** (0.04-0.07) |
| NPV | Baseline | **0.96** (0.93-0.98) | **0.97** (0.96-0.98) | **0.97** (0.95-0.98) | **0.96** (0.92-0.98) | **0.98** (0.96-0.99) | **0.98** (0.96-0.99) | **0.98** (0.97-0.99) | **0.98** (0.96-0.99) |
|  | ROS | **0.96** (0.91-0.98) | **0.97** (0.95-0.98) | **0.97** (0.96-0.97) | **0.96** (0.93-0.98) | **0.98** (0.96-0.99) | **0.98** (0.97-0.99) | **0.98** (0.96-0.99) | **0.98** (0.97-0.99) |
|  | RUS | **0.96** (0.92-0.98) | **0.97** (0.95-0.98) | **0.97** (0.95-0.97) | **0.96** (0.93-0.98) | **0.98** (0.97-0.99) | **0.98** (0.97-0.99) | **0.98** (0.96-0.99) | **0.98** (0.96-0.98) |
|  | SMOTEENN | **0.96** (0.94-0.98) | **0.97** (0.95-0.98) | **0.97** (0.95-0.98) | **0.96** (0.90-0.98) | **0.98** (0.97-0.99) | **0.98** (0.97-0.98) | **0.98** (0.96-0.98) | **0.98** (0.96-0.99) |

Abbreviations: ROS, RandomOverSampler; RUS, RandomUnderSampler; SMOTEENN, Synthetic Minority Over-sampling Technique with Edited Nearest Neighbors.

* Note: Baseline represents the training pipeline without resampling.
