# Supplementary material for: Admission photoplethysmography-based mortality prediction in hospitalized Ugandan children with suspected or confirmed infection: A feasibility study
Source: PLOS Digit Health. 2026 Aug 6;5(8):e0001056. doi: 10.1371/journal.pdig.0001056 (PMC13446674; doi:10.1371/journal.pdig.0001056)
Supplement: Supplementary file 6 [file pdig.0001056.s006.docx]

**S6 Appendix. Pairwise Nemenyi post-hoc comparisons.**

**Table 1. Pairwise Nemenyi post-hoc comparisons of AUROC and AUPRC from test folds, stratified by age cohort.**

|  | **0–6-month cohort** | | | | **6–60-month cohort** | | | |
| --- | --- | --- | --- | --- | --- | --- | --- | --- |
|  | **LR*_10_*** | **RF*_10_*** | **XGB*_10_*** | **MLP*_10_*** | **LR*_10_*** | **RF*_10_*** | **XGB*_10_*** | **MLP*_10_*** |
| **AUROC** | | | | | | | | |
| LR*_10_* | — | 0.00* | 0.01* | 0.96 | — | 0.01* | 0.06 | 0.90 |
| RF*_10_* | 0.00* | — | 0.32 | 0.00* | 0.01* | — | 0.93 | 0.01* |
| XGB*_10_* | 0.01* | 0.32 | — | 0.03* | 0.06 | 0.93 | — | 0.06 |
| MLP*_10_* | 0.96 | 0.00* | 0.03* | — | 1.00 | 0.01* | 0.06 | — |
| **AUPRC** | | | | | | | | |
| LR*_10_* | Friedman test found no significant differences. | | | | — | 1.00 | 0.01* | 0.98 |
| RF*_10_* |  |  |  |  | 1.00 | — | 0.03* | 1.00 |
| XGB*_10_* |  |  |  |  | 0.01* | 0.03* | — | 0.05* |
| MLP*_10_* |  |  |  |  | 0.98 | 1.00 | 0.05* | — |

Asterisks (*) denote statistically significant differences at the 0.05 significance level. Nemenyi post-hoc comparisons were conducted only when the Friedman test identified significant differences (p-value<0.05).
