# Supplementary material for: Admission photoplethysmography-based mortality prediction in hospitalized Ugandan children with suspected or confirmed infection: A feasibility study
Source: PLOS Digit Health. 2026 Aug 6;5(8):e0001056. doi: 10.1371/journal.pdig.0001056 (PMC13446674; doi:10.1371/journal.pdig.0001056)
Supplement: Supplementary file 7 [file pdig.0001056.s007.docx]

**S7 Appendix. SHAP summary and frequency plots.**

**Contents**

[Fig 1. SHAP and frequency plots of LR*_10_* obtained from test folds, stratified by age cohort.	2](#_Toc209798567)

[Fig 2. SHAP and frequency plots of RF*_10_* obtained from test folds, stratified by age cohort.	3](#_Toc209798568)

[Fig 3. SHAP and frequency plots of MLP*_10_* obtained from test folds, stratified by age cohort.	4](#_Toc209798569)

**Fig 1.** **SHAP and frequency plots of LR_10_ obtained from test folds, stratified by age cohort.**

**Fig 2. SHAP and frequency plots of RF_10_ obtained from test folds, stratified by age cohort.**

**Fig 3. SHAP and frequency plots of MLP_10_ obtained from test folds, stratified by age cohort.**
