# Supplementary material for: Admission photoplethysmography-based mortality prediction in hospitalized Ugandan children with suspected or confirmed infection: A feasibility study
Source: PLOS Digit Health. 2026 Aug 6;5(8):e0001056. doi: 10.1371/journal.pdig.0001056 (PMC13446674; doi:10.1371/journal.pdig.0001056)
Supplement: Supplementary file 8 [file pdig.0001056.s008.docx]

**S8 Appendix. Sensitivity analysis of early mortality outcomes.**

To explore whether admission PPG features also carried prognostic value for earlier mortality windows, additional exploratory analyses were conducted using the XGB classifier. XGB was selected because, in the main study, XGB_10_ and RF_10_ outperformed LR_10_ and MLP_10_, and XGB offered greater computational efficiency. A full re-development of all model families for multiple early mortality definitions was considered beyond the scope of the present study; therefore, analyses were restricted to XGB.

Two complementary procedures were performed for each cohort (0–6 months and 6–60 months). First, the original 10 PPG features selected in XGB_10_ were retained, and the 6×(5×5) nested CV pipeline without RFE was repeated using early mortality outcomes defined as death within 1 day and within 2 days of admission. Second, model development was repeated from scratch for these early mortality outcomes using feature sets of size 2–10. Across both cohorts, models using 6 features achieved near-optimal performance and are reported as XGB_6_.

Early mortality was infrequent (0–6 months: 1‑day 2.3%, 2‑day 3.4%; 6–60 months: 1‑day 1.6%, 2‑day 2.2%), and results should therefore be interpreted cautiously. In the 0–6‑month cohort, XGB_10_ achieved AUROC values of 0.70 for the 48‑hour outcome (the primary mortality outcome used in the main analysis), 0.73 for 1‑day mortality, and 0.70 for 2‑day mortality, with corresponding AUPRC values of 0.18, 0.12, and 0.13. In the 6–60‑month cohort, AUROC values were 0.67 (48‑hour outcome), 0.75 (1‑day), and 0.73 (2‑day), with AUPRC values of 0.14, 0.11, and 0.13. The re‑developed XGB_6_ models showed similar patterns. The results are summarized in Table 1.

Overall, these exploratory analyses suggest that admission PPG features retain prognostic value for early mortality, although precision-recall performance is constrained by the low prevalence of early deaths.

**Table 1. Exploratory early mortality prediction results using XGB, stratified by age cohort.**

|  | **0–6-month cohort** | | | | | **6–60-month cohort** | | | | |
| --- | --- | --- | --- | --- | --- | --- | --- | --- | --- | --- |
|  | **XGB*_10_*** | | | **XGB*_6_*** | | **XGB*_10_*** | | | **XGB*_6_*** | |
|  | *48-hour* | *1-day* | *2-day* | *1-day* | *2-day* | *48-hour* | *1-day* | *2-day* | *1-day* | *2-day* |
| AUROC | 0.70 | 0.73 | 0.70 | 0.69 | 0.68 | 0.67 | 0.75 | 0.73 | 0.71 | 0.71 |
| AUPRC | 0.18 | 0.12 | 0.13 | 0.09 | 0.10 | 0.14 | 0.11 | 0.13 | 0.09 | 0.11 |
| Sensitivity | 0.83 | 0.84 | 0.83 | 0.84 | 0.83 | 0.83 | 0.84 | 0.81 | 0.84 | 0.81 |
| Specificity | 0.42 | 0.49 | 0.43 | 0.46 | 0.41 | 0.38 | 0.54 | 0.51 | 0.49 | 0.45 |
| PPV | 0.10 | 0.04 | 0.05 | 0.04 | 0.05 | 0.05 | 0.03 | 0.04 | 0.03 | 0.04 |
| NPV | 0.97 | 0.99 | 0.99 | 0.99 | 0.98 | 0.98 | 0.99 | 0.99 | 0.99 | 0.99 |
