# Supplementary material for: Admission photoplethysmography-based mortality prediction in hospitalized Ugandan children with suspected or confirmed infection: A feasibility study
Source: PLOS Digit Health. 2026 Aug 6;5(8):e0001056. doi: 10.1371/journal.pdig.0001056 (PMC13446674; doi:10.1371/journal.pdig.0001056)
Supplement: Supplementary file 9 [file pdig.0001056.s010.docx]

| To the Editors,  *PLOS Digital Health* | May 16^th^, 2026 |
| --- | --- |

Dear Editors,

We thank you and the reviewers for the thoughtful and constructive comments on our manuscript. We have revised the manuscript accordingly and provide detailed point-by-point responses to each comment below. We appreciate the opportunity to improve our work and hope the revised version meets your expectations.

Sincerely,
Mahan Rahimi, on behalf of all co-authors

Reviewer #1

**Comment 1.1:** *One important limitation of the modelling work is the strong imbalance in the data. The models were naturally inclined to predict survival more often. The negative predictive value was very high, but precision and AUPRC remained low and only slightly better than what would be expected from chance based on outcome prevalence.*

**Response 1.1:** We have now clarified that class imbalance was formally evaluated and documented. During our initial model development phase, we addressed class imbalance by testing three commonly used resampling techniques, including RandomOverSampler, RandomUnderSampler, and SMOTEENN, to determine whether they could improve performance. These methods were integrated into the inner cross-validation loop and assessed across all classifiers and age cohorts. Our analyses showed that these resampling strategies did not yield meaningful improvements in discrimination or other performance metrics compared with our baseline approach, which is consistent with prior evidence that resampling does not always improve the performance of clinical risk prediction models under severe class imbalance [1]. Given these findings, we proceeded with the baseline models for our final analysis. We have now formally documented this evaluation in *Section 2.6.2 Model validation pipeline* and included the full summary of these comparative results in *S5 Appendix* to provide a more comprehensive view of our model development process.

**Comment 1.2:** *Another concern is that sensitivity was not reported. Without sensitivity and without strategies to deal with the imbalance, it is difficult to judge whether the models can meaningfully identify children truly at risk of death. To strengthen the work, revisions should consider handling the imbalance, reporting sensitivity, and using evaluation metrics that better match the intended purpose.*

**Response 1.2:** We agree that sensitivity is essential for a model intended to identify children at risk of death. In our original submission, threshold-dependent metrics (specificity, PPV, and NPV) were computed at a probability threshold chosen to ensure at least 80% sensitivity; thus, sensitivity was implicitly reflected in the reported results. To address the reviewer’s request for explicit reporting, we have now added sensitivity as a separate row in *Table 3*. Because our intended use case is screening, where false negatives are more consequential than false positives, we prioritized discrimination metrics and threshold-based metrics that reflect this objective. AUROC was particularly relevant because it summarizes ranking performance across the score range, including the low-threshold region needed to maintain high sensitivity, while AUPRC and PPV provide complementary information under severe imbalance. This is consistent with recent work showing that AUPRC is not inherently superior in imbalanced settings and that metric choice should depend on the deployment scenario [2]. In addition, as detailed in our response to Comment 1.1, we have incorporated a formal assessment of class imbalance handling strategies to ensure our results are robust.

Reviewer #2

This manuscript presents a feasibility study using admission photoplethysmography (PPG) features to predict in-hospital mortality in Ugandan children under five. Through a secondary analysis of Smart Discharges data, the authors trained machine learning models on 1-minute PPG recordings. This approach addresses a critical diagnostic gap in resource-limited settings.

The methodology is rigorous, employing nested cross-validation and comprehensive feature engineering. Whilst the models achieved moderate discrimination (AUROC ~0.67–0.70), Decision Curve Analysis (DCA) suggests they offer superior risk stratification compared to ‘treat-all’ or ‘treat-none’ strategies. However, specific methodological constraints require clarification.

**Major Comments**

**Comment 2.1:** *Outcome Definition (Lines 227–233): The authors classify patients who “discharged against medical advice” or “left without notice” as mortalities. This introduces potential bias. Please provide the specific number of these patients. If the figure is significant, a sensitivity analysis excluding this group (or treating them as survivors) is recommended to ensure the validity of the results.*

**Response 2.1:** We have clarified the outcome definition and provided the requested counts. The primary outcome—peri‑hospitalization mortality—includes deaths occurring in hospital, on the day of discharge, or within 48 hours post‑discharge. As described in the manuscript, the Smart Discharges project conducted active post‑discharge follow‑up through scheduled visits, and both in‑ and out‑of‑hospital deaths were systematically recorded. Children who left against medical advice or without notice were not classified as non‑survivors by default; only those with a confirmed death within 48 hours were included as outcome events. We have now added the exact counts for each mortality subtype to *Table 2*. These subgroups represent a small minority of all deaths:

- *0–6 months:* 149/170 in‑hospital (87.7%), 7/170 on discharge day (4.1%), 14/170 within 48 hours post‑discharge (8.2%);
- *6–60 months:* 129/142 in‑hospital (90.9%), 3/142 on discharge day (2.1%), 10/142 within 48 hours post‑discharge (7%).

Given that the vast majority of deaths occurred during hospitalization, and all early post‑discharge deaths were confirmed through the Smart Discharges follow‑up system, it is highly unlikely that reclassifying this group as survivors would materially alter model performance. We have also revised the *second limitation* in *Section 4. Discussion* to clarify that, although early post‑discharge outcomes were reliably ascertained in this dataset, similar assumptions may not hold in settings without active surveillance.

**Comment 2.2:** *Data Exclusion & Feasibility (Lines 511–516): Approximately 10% of subjects were excluded due to inadequate PPG recordings. In a clinical context, signal failure often correlates with poor perfusion found in severe sepsis. If the tool fails on the sickest children, the feasibility claim is compromised. Please compare the demographics and mortality rates of the excluded population against the included cohort to address this limitation.*

**Response 2.2:** As requested, we compared the excluded and included groups and updated the manuscript accordingly. At face value, the exclusion of children with inadequate PPG signals raises the possibility of selection bias. However, our comparative analysis showed that the main difference between the two groups was mortality, with excluded children exhibiting higher mortality, consistent with greater illness severity and impaired peripheral perfusion rather than random data loss or systematic bias. We have revised *Section 3.1 Study population* to report this finding, and we have also updated the *first limitation* in *Section 4. Discussion* to frame this as a practical feasibility constraint rather than an analytical limitation. Because our models are intended as admission-phase screening tools, they are designed for children in whom a usable signal can be obtained. Children too unwell for reliable measurement would require immediate management regardless of model output; thus, the inability to acquire a signal serves as an urgent clinical indicator in its own right. Baseline characteristics of the excluded population are provided in *S4 Appendix*.

**Comment 2.3:** *Recording Duration (Line 523): 1-minute recordings are suboptimal for frequency-domain Pulse Rate Variability (PRV) features (specifically VLF and LF). Given these were top predictors in the 6–60 month cohort, the authors must explicitly state that frequency-domain findings should be interpreted with caution. It would be valuable to note if time-domain features alone yield similar performance.*

**Response 2.3:** We acknowledge that 1-minute recordings are suboptimal for the reliable estimation of certain frequency-domain features and have updated the *third limitation* in *Section 4. Discussion* to explicitly state that these findings should be interpreted with caution. Regarding the suggestion to evaluate time-domain features alone, our current pipeline utilizes recursive feature elimination (RFE) to identify the optimal subset of predictors from all available candidate features. Because RFE selects the subset that maximizes model performance, the inclusion of frequency-domain features confirms that they provide predictive utility that complements time-domain indices. Restricting the analysis to time-domain features would likely result in diminished performance by excluding these informative predictors. Given that our current feature selection process inherently optimizes for predictive utility, we believe an additional analysis restricted to time-domain features is outside the scope of this study.

**Minor Comments**

**Comment 2.4:** *Fig. 3 (Performance Saturation): The analysis showing performance plateauing after ~10 features is excellent. It strongly supports the argument for feasibility regarding deployment on simpler edge devices.*

**Response 2.4:** We appreciate this feedback and thank the reviewer for highlighting this point.

**Comment 2.5:** *Precision-Recall: The low AUPRC (0.14–0.20) is expected given the low outcome prevalence. The authors’ focus on Negative Predictive Value (>97%) is the correct clinical framing for ruling out low-risk patients.*

**Response 2.5:** We appreciate this positive feedback and thank the reviewer for recognizing the clinical relevance of our framing.

**Comment 2.6:** *SpO_2_ & Pulse Rate: The finding that standard SpO_2_ and Pulse Rate were not dominant features compared to PRV indices (Lines 503–510) validates the use of advanced signal processing. This warrants greater emphasis in the conclusion.*

**Response 2.6:** This point has been addressed by strengthening the emphasis on advanced signal processing value. We have revised *Section 4. Discussion* to further highlight that the lower importance of SpO_2_ and pulse rate—despite their established clinical relevance—supports the added prognostic value of PPG‑derived variability indices. This finding reinforces the rationale for applying advanced signal processing approaches in pediatric sepsis risk stratification, and this emphasis has now been made more explicit in the revised text.

Reviewer #3

Overall, this is a well-executed and methodologically rigorous study addressing an important gap in pediatric triage in LMIC settings. The manuscript would be strengthened by clearer positioning of its clinical role, deeper consideration of bias and generalizability, and more explicit discussion of implementation trade-offs given modest discrimination and low PPV.

**Comments**

**Comment 3.1:** *Provide justification for analyzing <6 months and 6 to 59 months separately.*

**Response 3.1:** Thank you for this important question. We analyzed children <6 months and 6–60 months separately because these two cohorts represent distinct clinical and developmental stages, with different baseline risk profiles, causes of mortality, and physiological maturity. Infants <6 months are particularly vulnerable to conditions related to perinatal and early-life factors, whereas children 6–60 months have a different pattern of infectious and nutritional risks. This stratification therefore allowed us to capture age-specific prognostic patterns without masking differences across these clinically distinct groups.

**Comment 3.2:** *The study collected two one-minute PPG recordings, how were they included in the study? Was their median/mean used or how was only one reading selected for inclusion in the study?*

**Response 3.2:** We have clarified how the two recordings were handled and how features were summarized. We retained only the highest-quality recording (inadequacy score <0.01) from the two 1-minute PPG recordings per child, as described in Section 2.5.2 Signal quality thresholding. Pulse-level feature values within that selected recording were then summarized using the median, unless otherwise stated, as detailed in Section 2.5.3 Feature extraction.

**Comment 3.3:** *The authors excluded 274 which represents ~11% of those included. Were these excluded children any different from the 2,433 subjects who remained? Can the authors speculate the effects of missing these children in the model performance?*

**Response 3.3:** This point has been addressed by referring the reviewer to our detailed comparison in Response 2.2. As noted in Response 2.2, we compared the excluded and included groups and found that the primary difference was mortality rate, with excluded children experiencing higher mortality. This pattern indicates that inability to obtain a usable PPG signal reflects severe illness rather than random data loss. The implications for model performance and feasibility are discussed in detail in Response 2.2, and the corresponding clarification has been incorporated into the revised manuscript.

**Comment 3.4:** *Selecting the single highest-quality recording per child may overestimate real-world performance. Maybe the authors should discuss whether using the first recording or a randomly selected recording would alter results or run sensitivity analyses.*

**Response 3.4:** We appreciate the reviewer’s point regarding potential differences between selecting the highest‑quality recording versus using the first or a randomly chosen recording. Because this was a feasibility study and the Smart Discharges project collected dual recordings to increase the likelihood of obtaining at least one usable waveform, we used the higher‑quality trace to ensure that feature extraction was based on physiologically interpretable data. Sensitivity analyses using first or randomly selected recordings would help quantify the impact of quality‑based selection; however, such methodological comparisons fall outside the scope of the present feasibility study and are better suited to future work focused on acquisition‑strategy robustness.

**Comment 3.5:** *The inclusion of deaths within 48 hours post-discharge, especially among children discharged against medical advice could introduce some bias since you can’t establish their vital status after absconding hospital. Sensitivity analyses excluding post-discharge deaths or stratification by in-hospital vs. post-discharge mortality would help assess robustness.*

**Response 3.5:** This point has been addressed by referring the reviewer to our detailed clarification in Response 2.1. As summarized in Response 2.1, children who left against medical advice or without notice were not classified as deaths by default; only those with a confirmed death within 48 hours—ascertained through the Smart Discharges active follow‑up system—were included as outcome events. We have added the exact counts for each mortality subtype to Table 2 and clarified in Section 4. Discussion that early post‑discharge outcomes were reliably captured in this dataset. The implications for potential bias and robustness are discussed in detail in Response 2.1, and the corresponding clarification has been incorporated into the revised manuscript.

**Comment 3.6:** *The manuscript does not clearly state whether any techniques (e.g., class weighting, resampling) were used to address severe class imbalance. If none were applied, please justify this choice, as it can substantially affect AUPRC and PPV.*

**Response 3.6:** This point has been addressed by referring the reviewer to our detailed explanation in Response 1.1. As summarized in Response 1.1, we evaluated multiple imbalance handling strategies—including RandomOverSampler, RandomUnderSampler, and SMOTEENN—within the inner cross‑validation loop across all classifiers and age cohorts. These approaches did not yield meaningful improvements in discrimination or threshold‑dependent metrics compared with the baseline models. The rationale for proceeding without resampling and the corresponding updates to the manuscript are described in detail in Response 1.1.

**Interpretability claims**

**Comment 3.7:** *While SHAP improves interpretability, many PPG-derived features are not clinically intuitive. The authors should clarify how frontline clinicians are expected to act on model outputs, given the abstract nature of many predictors.*

**Response 3.7:** We thank the reviewer for raising this important point. The models developed in this study are intended as screening tools, not diagnostic aids, and therefore frontline users are not expected to interpret individual PPG‑derived features. Clinical or operational action is guided by the predicted risk category, which supports early identification of children who may require closer monitoring or escalation of care. This design aligns with the intended use of the tools in resource‑limited settings, where admission‑time assessments may be performed by minimally trained staff using rapid, non‑invasive measurements. SHAP was incorporated to enhance model‑level transparency, allowing researchers and implementers to understand which physiological domains contribute most to risk prediction, even if some features are not directly interpretable at the bedside. This information is valuable for future model refinement, feature selection, and understanding the physiological signals captured by the PPG waveform, rather than for guiding point‑of‑care clinical decisions.

**Comment 3.8:** *Mortality risk prediction at admission may be more clinically relevant for early mortality (e.g., 24–48 hours) than for peri-hospitalization mortality. The authors acknowledge this limitation, but can they explicitly explore the analyses if data allow.*

**Response 3.8:** Thank you for this helpful suggestion. To explore whether admission PPG features also carried prognostic value for earlier mortality windows, additional exploratory analyses were conducted using the XGB classifier. A full re-development of all model families for multiple earlier mortality windows was considered beyond the scope of the present study; therefore, analyses were restricted to XGB, which demonstrated strong performance and computational efficiency in the main results of the manuscript. Two analyses were performed for each cohort (0–6 months and 6–60 months): (i) the original 10-feature model was re-evaluated for mortality within 1 day and within 2 days of admission, and (ii) model development was repeated from scratch for these outcomes using feature sets of size 2–10. Across both cohorts, early mortality was infrequent, but AUROC values for 1-day and 2-day mortality were comparable to those for the primary 48-hour outcome, and models using 6 features achieved near-optimal performance. These findings suggest that the admission PPG features retain prognostic value for earlier mortality windows, although precision-recall performance is limited by the low event rates. The full results have been added to S8 Appendix, and the fourth limitation in Section 4. Discussion has been updated to reference this exploratory analysis.

**Comment 3.9:** *The study included many hospitals. How was clustering across these health facility handled in the model?*

**Response 3.9:** We have clarified how data from multiple hospitals were handled in the modeling pipeline. Given the limited sample size per hospital and our goal of developing a generalizable model for diverse Ugandan settings, we pooled data across all hospitals within each cohort. This approach maximizes statistical power and follows standard practice in multi‑center studies where site‑specific models would be underpowered. We have updated Section 2.6.2 Model validation pipeline to state this explicitly.

**Comment 3.10:** *The manuscript alternates between “peri-hospitalization mortality” and “hospital mortality.” Please stick to one term.*

**Response 3.10:** We thank the reviewer for noting this point. We have ensured consistent terminology throughout the manuscript by using “hospital mortality” in all sections except Section 2.6.1 Outcome definition, where “peri‑hospitalization mortality” is explicitly defined.

**References**

| 1. | Ke JXC, DhakshinaMurthy A, George RB, Branco P. The effect of resampling techniques on the performances of machine learning clinical risk prediction models in the setting of severe class imbalance: development and internal validation in a retrospective cohort. Discov Artif Intell. 2024; 4(1): 91. |
| --- | --- |
| 2. | McDermott M, Zhang H, Hansen LH, Angelotti G, Gallifant J. A closer look at AUROC and AUPRC under class imbalance. In Advances in Neural Information Processing Systems; 2024. p. 44102-44163. |
