# Supplementary material for: Admission photoplethysmography-based mortality prediction in hospitalized Ugandan children with suspected or confirmed infection: A feasibility study
Source: PLOS Digit Health. 2026 Aug 6;5(8):e0001056. doi: 10.1371/journal.pdig.0001056 (PMC13446674; doi:10.1371/journal.pdig.0001056)
Supplement: Supplementary file 10 [file pdig.0001056.s001.docx]

**S1 Appendix. Preprocessed PPG waveform.**

**Fig 1. Preprocessing steps of a PPG waveform.**
