# Supplementary material for: Admission photoplethysmography-based mortality prediction in hospitalized Ugandan children with suspected or confirmed infection: A feasibility study
Source: PLOS Digit Health. 2026 Aug 6;5(8):e0001056. doi: 10.1371/journal.pdig.0001056 (PMC13446674; doi:10.1371/journal.pdig.0001056)
Supplement: Peer review history

# Peer review: Decision Letter 0

**DOI:** 10.1371/journal.pdig.0001056.r001

**Authors:** Peter H Charlton, John Batani

9 Feb 2026

PDIG-D-25-00827

Admission photoplethysmography-based mortality prediction in hospitalized Ugandan children with suspected or confirmed infection: a feasibility study

PLOS Digital Health

Dear Dr. Rahimi,

Thank you for submitting your manuscript to PLOS Digital Health. After careful consideration, we feel that it has merit but does not fully meet PLOS Digital Health's publication criteria as it currently stands. Therefore, we invite you to submit a revised version of the manuscript that addresses the points raised during the review process.

Please submit your revised manuscript by Apr 10 2026 11:59PM. If you will need more time than this to complete your revisions, please reply to this message or contact the journal office at digitalhealth@plos.org. When you're ready to submit your revision, log on to https://www.editorialmanager.com/pdig/ and select the 'Submissions Needing Revision' folder to locate your manuscript file.

Please include the following items when submitting your revised manuscript:

* A letter that responds to each point raised by the editor and reviewer(s). You should upload this letter as a separate file labeled 'Response to Reviewers'. This file does not need to include responses to any formatting updates and technical items listed in the 'Journal Requirements' section below.

* A marked-up copy of your manuscript that highlights changes made to the original version. You should upload this as a separate file labeled 'Revised Manuscript with Track Changes'.

* An unmarked version of your revised paper without tracked changes. You should upload this as a separate file labeled 'Manuscript'.

If you would like to make changes to your financial disclosure, competing interests statement, or data availability statement, please make these updates within the submission form at the time of resubmission. Guidelines for resubmitting your figure files are available below the reviewer comments at the end of this letter.

We look forward to receiving your revised manuscript.

Kind regards,

John Batani

Academic Editor

PLOS Digital Health

Peter H Charlton

Section Editor

PLOS Digital Health

Leo Anthony Celi

Editor-in-Chief

PLOS Digital Health

orcid.org/0000-0001-6712-6626

**Journal Requirements:**

1. Please ensure that your Ethics Statement is available in its entirety at the beginning of your Methods section, under a subheading 'Ethics Statement'.

If the reviewer comments include a recommendation to cite specific previously published works, please review and evaluate these publications to determine whether they are relevant and should be cited. There is no requirement to cite these works unless the editor has indicated otherwise.

**Additional Editor Comments (if provided):**

Based on the review comments, I invite you to revise the manuscript

[Note: HTML markup is below. Please do not edit.]

**Reviewers' Comments:**

Reviewer's Responses to Questions

**Comments to the Author**

1. Does this manuscript meet PLOS Digital Health’s publication criteria? Is the manuscript technically sound, and do the data support the conclusions? The manuscript must describe methodologically and ethically rigorous research with conclusions that are appropriately drawn based on the data presented.

Reviewer #1: Yes

Reviewer #2: Yes

Reviewer #3: Yes

**********

2. Has the statistical analysis been performed appropriately and rigorously?

Reviewer #1: No

Reviewer #2: Yes

Reviewer #3: Yes

**********

3. Have the authors made all data underlying the findings in their manuscript fully available (please refer to the Data Availability Statement at the start of the manuscript PDF file)?

The PLOS Data policy requires authors to make all data underlying the findings described in their manuscript fully available without restriction, with rare exception. The data should be provided as part of the manuscript or its supporting information, or deposited to a public repository. For example, in addition to summary statistics, the data points behind means, medians and variance measures should be available. If there are restrictions on publicly sharing data—e.g. participant privacy or use of data from a third party—those must be specified.

Reviewer #1: No

Reviewer #2: Yes

Reviewer #3: Yes

**********

4. Is the manuscript presented in an intelligible fashion and written in standard English?

PLOS Digital Health does not copyedit accepted manuscripts, so the language in submitted articles must be clear, correct, and unambiguous. Any typographical or grammatical errors should be corrected at revision, so please note any specific errors here.

Reviewer #1: Yes

Reviewer #2: Yes

Reviewer #3: Yes

**********

5. Review Comments to the Author

Please use the space provided to explain your answers to the questions above. You may also include additional comments for the author, including concerns about dual publication, research ethics, or publication ethics. (Please upload your review as an attachment if it exceeds 20,000 characters)

Reviewer #1: One important limitation of the modelling work is the strong imbalance in the data. The models were naturally inclined to predict survival more often. The negative predictive value was very high, but precision and AUPRC remained low and only slightly better than what would be expected from chance based on outcome prevalence.

Another concern is that sensitivity was not reported. Without sensitivity and without strategies to deal with the imbalance, it is difficult to judge whether the models can meaningfully identify children truly at risk of death. To strengthen the work, revisions should consider handling the imbalance, reporting sensitivity, and using evaluation metrics that better match the intended purpose.

Reviewer #2: This manuscript presents a feasibility study using admission photoplethysmography (PPG) features to predict in-hospital mortality in Ugandan children under five. Through a secondary analysis of Smart Discharges data, the authors trained machine learning models on 1-minute PPG recordings. This approach addresses a critical diagnostic gap in resource-limited settings.

The methodology is rigorous, employing nested cross-validation and comprehensive feature engineering. Whilst the models achieved moderate discrimination (AUROC ~0.67–0.70), Decision Curve Analysis (DCA) suggests they offer superior risk stratification compared to ‘treat-all’ or ‘treat-none’ strategies. However, specific methodological constraints require clarification.

Major Comments

Outcome Definition (Lines 227–233): The authors classify patients who "discharged against medical advice" or "left without notice" as mortalities. This introduces potential bias. Please provide the specific number of these patients. If the figure is significant, a sensitivity analysis excluding this group (or treating them as survivors) is recommended to ensure the validity of the results.

Data Exclusion & Feasibility (Lines 511–516): Approximately 10% of subjects were excluded due to inadequate PPG recordings. In a clinical context, signal failure often correlates with poor perfusion found in severe sepsis. If the tool fails on the sickest children, the feasibility claim is compromised. Please compare the demographics and mortality rates of the excluded population against the included cohort to address this limitation.

Recording Duration (Line 523): 1-minute recordings are suboptimal for frequency-domain Pulse Rate Variability (PRV) features (specifically VLF and LF). Given these were top predictors in the 6–60 month cohort, the authors must explicitly state that frequency-domain findings should be interpreted with caution. It would be valuable to note if time-domain features alone yield similar performance.

Minor Comments

Fig. 3 (Performance Saturation): The analysis showing performance plateauing after ~10 features is excellent. It strongly supports the argument for feasibility regarding deployment on simpler edge devices.

Precision-Recall: The low AUPRC (0.14–0.20) is expected given the low outcome prevalence. The authors’ focus on Negative Predictive Value (>97%) is the correct clinical framing for ruling out low-risk patients.

SpO2 & Pulse Rate: The finding that standard SpO2 and Pulse Rate were not dominant features compared to PRV indices (Lines 503–510) validates the use of advanced signal processing. This warrants greater emphasis in the conclusion.

Reviewer #3: Overall, this is a well-executed and methodologically rigorous study addressing an important gap in pediatric triage in LMIC settings. The manuscript would be strengthened by clearer positioning of its clinical role, deeper consideration of bias and generalizability, and more explicit discussion of implementation trade-offs given modest discrimination and low PPV.

Comments

-Provide justification for analyzing <6 months and 6 to 59 months separately.

-The study collected two one-minute PPG recordings, how were they included in the study? Was their median/mean used or how was only one reading selected for inclusion in the study?

-The authors excluded 274 which represents ~ 11% of those included. Were these excluded children any different from the 2,433 subjects who remained? Can the authors speculate the effects of missing these children in the model performance

-Selecting the single highest-quality recording per child may overestimate real-world performance. Maybe the authors should discuss whether using the first recording or a randomly selected recording would alter results or run sensitivity analyses.

-The inclusion of deaths within 48 hours post-discharge, especially among children discharged against medical advice could introduce some bias since you can establish their vital status after absconding hospital

-Sensitivity analyses excluding post-discharge deaths or stratification by in-hospital vs. post-discharge mortality would help assess robustness.

-The manuscript does not clearly state whether any techniques (e.g., class weighting, resampling) were used to address severe class imbalance. If none were applied, please justify this choice, as it can substantially affect AUPRC and PPV.

Interpretability claims

-While SHAP improves interpretability, many PPG-derived features are not clinically intuitive. The authors should clarify how frontline clinicians are expected to act on model outputs, given the abstract nature of many predictors.

-Mortality risk prediction at admission may be more clinically relevant for early mortality (e.g., 24–48 hours) than for peri-hospitalization mortality. The authors acknowledge this limitation, but can they explicitly explore the analyses if data allow.

-The study included many hospitals. How was clustering across these health facility handled in the model?

-The manuscript alternates between “peri-hospitalization mortality” and “hospital mortality.” Please stick to one term.

**********

6. PLOS authors have the option to publish the peer review history of their article (what does this mean?). If published, this will include your full peer review and any attached files.

**Do you want your identity to be public for this peer review?**  If you choose “no”, your identity will remain anonymous but your review may still be made public.

For information about this choice, including consent withdrawal, please see our Privacy Policy.

Reviewer #1: No

Reviewer #2: **Yes:** Attlee Munyaradzi GAMUNDANI

Reviewer #3: **Yes:** Dr. Moses Ngari

**********

[NOTE: If reviewer comments were submitted as an attachment file, they will be attached to this email and accessible via the submission site. Please log into your account, locate the manuscript record, and check for the action link "View Attachments". If this link does not appear, there are no attachment files.]

**Figure resubmission:**

While revising your submission, we strongly recommend that you use PLOS’s NAAS tool (https://ngplosjournals.pagemajik.ai/artanalysis) to test your figure files. NAAS can convert your figure files to the TIFF file type and meet basic requirements (such as print size, resolution), or provide you with a report on issues that do not meet our requirements and that NAAS cannot fix.

After uploading your figures to PLOS’s NAAS tool - https://ngplosjournals.pagemajik.ai/artanalysis, NAAS will process the files provided and display the results in the "Uploaded Files" section of the page as the processing is complete. If the uploaded figures meet our requirements (or NAAS is able to fix the files to meet our requirements), the figure will be marked as "fixed" above. If NAAS is unable to fix the files, a red "failed" label will appear above. When NAAS has confirmed that the figure files meet our requirements, please download the file via the download option, and include these NAAS processed figure files when submitting your revised manuscript.

**Reproducibility:**

To enhance the reproducibility of your results, we recommend that authors of applicable studies deposit laboratory protocols in protocols.io, where a protocol can be assigned its own identifier (DOI) such that it can be cited independently in the future. Additionally, PLOS ONE offers an option to publish peer-reviewed clinical study protocols. Read more information on sharing protocols at https://plos.org/protocols?utm_medium=editorial-email&utm_source=authorletters&utm_campaign=protocols

# Author comment: Author response to Decision Letter 1

**DOI:** 10.1371/journal.pdig.0001056.r002

17 May 2026

Attachment Submitted filename: Response to Reviewers.docx

# Peer review: Decision Letter 1

**DOI:** 10.1371/journal.pdig.0001056.r003

**Authors:** Peter H Charlton, John Batani

13 Jul 2026

Admission photoplethysmography-based mortality prediction in hospitalized Ugandan children with suspected or confirmed infection: A feasibility study

PDIG-D-25-00827R1

Dear Ms. Rahimi,

We are pleased to inform you that your manuscript 'Admission photoplethysmography-based mortality prediction in hospitalized Ugandan children with suspected or confirmed infection: A feasibility study' has been provisionally accepted for publication in PLOS Digital Health.

Before your manuscript can be formally accepted you will need to complete some formatting changes, which you will receive in a follow-up email from a member of our team.

Please note that your manuscript will not be scheduled for publication until you have made the required changes, so a swift response is appreciated.

IMPORTANT: The editorial review process is now complete. PLOS will only permit corrections to spelling, formatting or significant scientific errors from this point onwards. Requests for major changes, or any which affect the scientific understanding of your work, will cause delays to the publication date of your manuscript.

If your institution or institutions have a press office, please notify them about your upcoming paper to help maximize its impact. If they'll be preparing press materials, please inform our press team as soon as possible -- no later than 48 hours after receiving the formal acceptance. Your manuscript will remain under strict press embargo until 2 pm Eastern Time on the date of publication. For more information, please contact digitalhealth@plos.org.

Thank you again for supporting Open Access publishing; we are looking forward to publishing your work in PLOS Digital Health.

Best regards,

John Batani, Ph.D

Academic Editor

PLOS Digital Health

***********************************************************

**Additional Editor Comments (if provided):**

**Reviewer Comments (if any, and for reference):**

Reviewer's Responses to Questions

**Comments to the Author**

1. If the authors have adequately addressed your comments raised in a previous round of review and you feel that this manuscript is now acceptable for publication, you may indicate that here to bypass the “Comments to the Author” section, enter your conflict of interest statement in the “Confidential to Editor” section, and submit your "Accept" recommendation.

Reviewer #3: All comments have been addressed

Reviewer #4: All comments have been addressed

**********

2. Does this manuscript meet PLOS Digital Health’s publication criteria? Is the manuscript technically sound, and do the data support the conclusions? The manuscript must describe methodologically and ethically rigorous research with conclusions that are appropriately drawn based on the data presented.

Reviewer #3: Yes

Reviewer #4: (No Response)

**********

3. Has the statistical analysis been performed appropriately and rigorously?

Reviewer #3: Yes

Reviewer #4: (No Response)

**********

4. Have the authors made all data underlying the findings in their manuscript fully available (please refer to the Data Availability Statement at the start of the manuscript PDF file)?

The PLOS Data policy requires authors to make all data underlying the findings described in their manuscript fully available without restriction, with rare exception. The data should be provided as part of the manuscript or its supporting information, or deposited to a public repository. For example, in addition to summary statistics, the data points behind means, medians and variance measures should be available. If there are restrictions on publicly sharing data—e.g. participant privacy or use of data from a third party—those must be specified.

Reviewer #3: Yes

Reviewer #4: (No Response)

**********

5. Is the manuscript presented in an intelligible fashion and written in standard English?

PLOS Digital Health does not copyedit accepted manuscripts, so the language in submitted articles must be clear, correct, and unambiguous. Any typographical or grammatical errors should be corrected at revision, so please note any specific errors here.

Reviewer #3: Yes

Reviewer #4: (No Response)

**********

6. Review Comments to the Author

Please use the space provided to explain your answers to the questions above. You may also include additional comments for the author, including concerns about dual publication, research ethics, or publication ethics. (Please upload your review as an attachment if it exceeds 20,000 characters)

Reviewer #3: Thank for addressing all my comments.

Reviewer #4: (No Response)

**********

7. PLOS authors have the option to publish the peer review history of their article (what does this mean?). If published, this will include your full peer review and any attached files.

**Do you want your identity to be public for this peer review?**  If you choose “no”, your identity will remain anonymous but your review may still be made public.

For information about this choice, including consent withdrawal, please see our Privacy Policy.

Reviewer #3: **Yes:** Dr Moses Ngari

Reviewer #4: None

**********
